# Investigating Patients' Continuance Intention Toward Conversational Agents in Outpatient Departments: Cross-sectional Field Survey

**DOI:** 10.2196/40681

**Published:** 2022-11-07

**Authors:** Xingyi Li, Shirong Xie, Zhengqiang Ye, Shishi Ma, Guangjun Yu

**Affiliations:** 1 School of Public Health Shanghai Jiao Tong University School of Medicine Shanghai China; 2 Eye and Ear, Nose, and Throat Hospital Fudan University Shanghai China; 3 Ruijin Hospital Shanghai Jiao Tong University School of Medicine Shanghai China; 4 Shanghai Children’s Hospital Shanghai Jiao Tong University School of Medicine Shanghai China

**Keywords:** conversational agent, continuance intention, expectation-confirmation model, partial least squares, structural equation modeling, chatbot, virtual assistant, cross-sectional, field study, optimization, outpatient, interview, qualitative, questionnaire, satisfaction, perceived usefulness, intention, adoption, attitude, perception

## Abstract

**Background:**

Conversational agents (CAs) have been developed in outpatient departments to improve physician-patient communication efficiency. As end users, patients’ continuance intention is essential for the sustainable development of CAs.

**Objective:**

The aim of this study was to facilitate the successful usage of CAs by identifying key factors influencing patients’ continuance intention and proposing corresponding managerial implications.

**Methods:**

This study proposed an extended expectation-confirmation model and empirically tested the model via a cross-sectional field survey. The questionnaire included demographic characteristics, multiple-item scales, and an optional open-ended question on patients’ specific expectations for CAs. Partial least squares structural equation modeling was applied to assess the model and hypotheses. The qualitative data were analyzed via thematic analysis.

**Results:**

A total of 172 completed questionaries were received, with a 100% (172/172) response rate. The proposed model explained 75.5% of the variance in continuance intention. Both satisfaction (β=.68; *P*<.001) and perceived usefulness (β=.221; *P*=.004) were significant predictors of continuance intention. Patients' extent of confirmation significantly and positively affected both perceived usefulness (β=.817; *P*<.001) and satisfaction (β=.61; *P*<.001). Contrary to expectations, perceived ease of use had no significant impact on perceived usefulness (β=.048; *P*=.37), satisfaction (β=−.004; *P*=.63), and continuance intention (β=.026; *P*=.91). The following three themes were extracted from the 74 answers to the open-ended question: personalized interaction, effective utilization, and clear illustrations.

**Conclusions:**

This study identified key factors influencing patients’ continuance intention toward CAs. Satisfaction and perceived usefulness were significant predictors of continuance intention (*P*<.001 and *P*<.004, respectively) and were significantly affected by patients’ extent of confirmation (*P*<.001 and *P*<.001, respectively). Developing a better understanding of patients’ continuance intention can help administrators figure out how to facilitate the effective implementation of CAs. Efforts should be made toward improving the aspects that patients reasonably expect CAs to have, which include personalized interactions, effective utilization, and clear illustrations.

## Introduction

### Background

Tertiary hospitals in China are occupied with many outpatients every day, which results in long waiting times and limited physician-patient communication during consultations. This phenomenon is caused mainly by two aspects. First, the large population base has resulted in a growing demand for medical services. Second, physicians have to finish both consulting patients and filling out medical histories during a limited amount of time. A study found that in the consultation room, 66.5% of physicians’ time was spent on communication and the examination of patients, and 20.7% of their time was spent on writing medical records [1]. Long waiting times, together with limited consultation times, further result in insufficient physician-patient communication and an incomplete understanding of conditions and diagnoses (ie, knowing all of the facts) [2,3]. Besides, during the ongoing COVID-19 pandemic, long waiting times have also put patients at risk for cross-infection [4].

Under national policies on the digital transformation of the health care industry, Shanghai, as a leading digital city, developed conversational agents (CAs) in outpatient departments, hoping to alleviate patient overload and improve communication efficiency. CAs are artificial intelligence programs that engage in dialogues with patients on mobile devices [5]. With these contextual question–answering agents, data on patients’ symptoms and medical histories can be captured and delivered to physicians’ workstations in structured forms before a consultation. During face-to-face consultations, physicians can rapidly gain an understanding of patients' general conditions and focus on other responsibilities [6], which has resulted in a man-machine integrated consultation model.

Prior studies have indicated that CAs can save time by reducing the time required for history taking, improve consultation efficiency, and enhance the completeness and accuracy of medical histories [7-10]. However, their potential has not been fully exploited, as the usage of CAs is often limited; 6 months after tertiary hospitals in Shanghai established CAs, the usage rates fell short of expectations (26% and 20%, respectively, for the second- and fourth-ranked hospitals). As end users, patients’ continuance intention is essential for the sustainable development of CAs [11], yet limited studies are available.

Based on the abovementioned research background and motivations, this study has 3 aims. First, it attempts to identify key factors influencing users’ continuance usage intention via a theoretical model. Second, it empirically examines the applicability of the model in the context of implementing CAs in outpatient departments. Third, it proposes corresponding managerial implications based on the results.

### Theoretical Background and Model

The usage of information systems includes the following two stages: preacceptance (acceptance before a system’s initial use) and postacceptance (acceptance after a system’s initial use; ie, continuance).

Even though initial use is an important first step toward realizing information systems’ success, it is mostly influenced by secondhand information from referent others or popular media rather than users’ actual interactions with the information system. In contrast, continuance after a system’s initial use is more realistic and unbiased, since it is grounded in users' firsthand experiences [12]. Therefore, the long-term viability and eventual success of information systems depend on users’ continued use.

There has been a considerable body of theory-based research on information system use in recent years. Among the theoretical models, the Technology Acceptance Model (TAM) is commonly applied to understand the initial acceptance of information systems, including intelligent health service systems (eg, registration systems and patient portals) [13-16]. The TAM predicts users' initial use of information systems based on the following two constructs: perceived usefulness and perceived ease of use [17].

To understand users’ continuance behavior in an information system context, an expectation-confirmation model (ECM) of information system continuance was proposed by Bhattacherjee [11]. This ECM has been empirically tested in a variety of contexts, including health services such as e-appointment systems and teleconsultations. The model predicts users' continuance intention via the following three antecedent constructs: perceived usefulness, confirmation, and satisfaction.

The ECM only incorporated perceived usefulness from the TAM, as Bhattacherjee [11] considered it to be the more salient and consistent predictor of information system use intention. However, both perceived usefulness and perceived ease of use are the primary motivators of information system acceptance in the TAM [18,19]. The significant impact of perceived ease of use on both perceived usefulness and usage intention has been verified in previous research (eg, studies on electronic health record acceptance by physicians and self-management technology acceptance by patients [20,21]). Given the particularities of patients and medical professionalism, perceived ease of use might also have the potential to influence patients’ continuance intention. Therefore, this study extended existing ECM constructs by integrating perceived ease of use, hoping to provide a better understanding of patients' continuance intention in the context of CAs.

**Figure 1:**
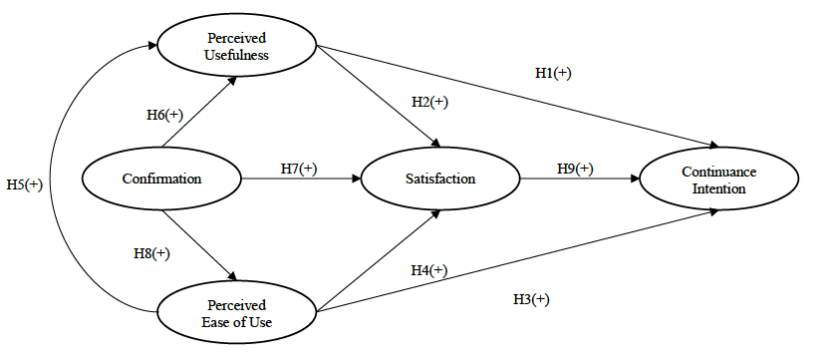
Research model. H: hypothesis; +: positive effect.

Based on the abovementioned theoretical reasoning and the results of previous research, we propose the following theoretical model (Figure 1) and hypotheses: (1) perceived usefulness positively affects continuance intention (hypothesis 1), (2) perceived usefulness positively affects satisfaction (hypothesis 2), (3) perceived ease of use positively affects continuance intention (hypothesis 3), (4) perceived ease of use positively affects satisfaction (hypothesis 4), (5) perceived ease of use positively affects perceived usefulness (hypothesis 5), (6) the confirmation of initial expectations positively affects perceived usefulness (hypothesis 6), (7) the confirmation of initial expectations positively affects satisfaction (hypothesis 7), (8) the confirmation of initial expectations positively affects perceived ease of use (hypothesis 8), and (9) satisfaction positively affects continuance intention (hypothesis 9).

## Methods

### Study Design and Setting

Tertiary hospitals in Shanghai established CAs in early 2021 to improve medical service efficiency and alleviate the overload in outpatient departments. Before face-to-face consultations, patients can provide their symptoms and medical histories on mobile devices via a contextual question–answering agent. Afterward, physicians can rapidly gain an understanding of patients' general conditions with the structured information delivered by CAs.

Shanghai Eye and ENT (ear, nose, and throat) Hospital has been a pioneer during the CA implementation process. Empirical data for this research were collected via a cross-sectional field survey that was conducted in the outpatient department of Shanghai Eye and ENT Hospital. The duration of this study was 3 months (November 2021 to January 2022). We invited patients and their companions who had used CAs. The survey was conducted near the pharmacy to make sure that patients finished their face-to-face consultations and minimize possible inconveniences.

### Sample Size and Sampling

The minimum sample size for this research was 124, and according to Marcoulides and Saunders [22], the minimum sample size depends on the maximum number of arrows pointing at a latent variable. Hoyle [23] recommended a sample size of 100 to 200 when performing path modeling. The convenience sampling technique was used, and a total of 172 questionnaires were completed. This sample size met the requirements for obtaining sufficient statistical power.

### Measurement Tools

The questionnaire included 3 parts—demographic characteristics, multiple-item scales, and the following optional open-ended question: “What are your expectations that CAs failed to meet?” The five constructs in the proposed model were measured by using multiple-item scales that were adapted from Davis [17] and Bhattacherjee [11], and the items were reworded to accommodate the context of CA use. Satisfaction items were scored on 5-point semantic differential scales. The remaining items were scored on 5-point Likert scales that ranged from 1 (strongly disagree) to 5 (strongly agree). Table 1 provides definitions and sources for the five constructs. The scale items were translated from English to Chinese because the survey was conducted in China. To avoid wording-related misapprehension, we used a back-translation process [24]. Multimedia Appendix 1 presents the items for each construct and their sources. A pretest was conducted among 25 patients to ensure the reliability and validity of the questionnaire.

**Table 1:** Definitions of constructs.

Construct	Operational definition	Reference
Continuance intention	Patients’ intention to continue using conversational agents	Bhattacherjee [11]
Satisfaction	Patients’ affects (feelings) prior to using conversational agents	Bhattacherjee [11]
Perceived usefulness	Patients’ perceptions of the expected benefits of conversational agents	Bhattacherjee [11]
Perceived ease of use	The degree to which patients believe that using conversational agents would be free from effort	Davis [17]
Confirmation	Patients’ perceptions of the congruence between expectations for conversational agents and their actual performance	Bhattacherjee [11]

### Data Collection

To help patients better understand their choices and remain focused, two postgraduates from Shanghai Jiao Tong University School of Medicine conducted the in-person survey, using paper questionnaires. During this process, the investigators read all of the questions aloud to the patients and filled in the questionnaire with their answers, which saved patients the trouble of reading the items themselves. The questionnaires ended with an optional open-ended question (“What are your expectations that CAs failed to meet?”). The answers were collected through brief interviews and written down in the form of detailed summaries by the investigators. A total of 172 valid questionnaires were collected, with a 100% (172/172) response rate, and 74 participants answered the optional open-ended question.

### Data Analysis

Descriptive statistics were performed by using SPSS 25.0 (IBM Corporation). A partial least squares structural equation model (PLS-SEM) analysis was performed in SmartPLS 3.3.3 (SmartPLS GmbH) to validate the research model and test the research hypotheses. A PLS-SEM was chosen because it is capable of producing robust results with restricted sample sizes and data lacking normality [25].

The implementation of this method was performed in 2 steps [26]. The first step consisted of assessing the reliability and validity of the measurement model using the partial least squares algorithm, while the second step focused on assessing the fit of the structural model and the significance of the hypotheses by using bootstrapping (5000 bootstrap samples) [27].

The qualitative data were analyzed via thematic analysis. Thematic analysis is a method for analyzing qualitative data that entails searching across a data set to identify, analyze, and report repeated patterns [28]. The initial codes were generated by deductively reading the manuscripts. This was done by a single coder, and the codes were reviewed by a second analyst [29]. After they reached consensus on the initial codes, the themes were extracted from and defined on the basis of the codes through group discussions.

### Ethics Approval

This study was approved by Shanghai Children’s Hospital (approval number: 2022R092-E01). All respondents participated in this study voluntarily and anonymously on the basis of informed consent.

## Results

### Demographic Information

A total of 172 questionnaires were complete and valid, with a 100% (172/172) response rate. The demographic information of CA users is listed in Table 2. Notably, 54.1% of the respondents were in the 25 to 35 years age group, and 63.4% (109/172) were women.

**Table 2:** Demographic information.

Participant characteristics		Participants (N=172), n (%)
**Gender**		
	Men	63 (36.6)
	Women	109 (63.4)
**Age (years)**		
	<25	12 (7)
	25-35	93 (54.1)
	36-45	46 (26.7)
	>45	21 (12.2)
**Relationship with the patient**		
	Patients themselves	106 (61.6)
	Patients’ children	22 (12.8)
	Patients’ parents	44 (25.6)
**Visit type**		
	First visit	112 (65.1)
	Return visit	60 (34.9)
**Number of visits over the past half year**		
	1	101 (58.7)
	2-3	47 (27.3)
	>3	24 (14)
**Number of times that a participant used a conversational agent**		
	1	136 (79.1)
	2-3	31 (18)
	>3	5 (2.9)

### Measurement Model Assessment

The measurement model was assessed in terms of construct reliability, convergent validity, and discriminative validity by performing a confirmatory composite analysis. The results are displayed in Table 3 and Table 4.

Reliability can be evaluated with Cronbach α and composite reliability values [30]. Convergent validity can be accessed with factor loading and average variance extracted (AVE) values [27,31]. As shown in Table 2, all of the Cronbach α and composite reliability values were above 0.7, the AVE for each construct was above 0.5, and the factor loadings for each item were above 0.7, indicating good reliability and convergent validity [27].

Discriminant validity reflects the extent to which constructs are significantly different from each other. To achieve discriminant validity, the square root of the AVE for a given construct must be higher than that construct’s correlation with other constructs, and this must hold true for all constructs [31]. As shown in Table 4, the results indicated that discriminate validity was achieved. Therefore, we concluded that the quality of the measurement model was sufficient for testing the hypotheses in the model.

**Table 3:** Construct reliability and convergent validity.

Constructs and items			Factor loadings		Cronbach α		CR^a^		AVE^b^
**CONF^c^**					.938		0.960		0.890
	CONF1	0.947							
	CONF2	0.940							
	CONF3	0.943							
**CI^d^**					.993		0.996		0.993
	CI1	0.996							
	CI2	0.996							
**PEOU^e^**					.792		0.871		0.694
	PEOU1	0.866							
	PEOU2	0.763							
	PEOU3	0.865							
**PU^f^**					.793		0.879		0.710
	PU1	0.825							
	PU2	0.761							
	PU3	0.933							
**SAT^g^**					.959		0.980		0.960
	SAT1	0.980							
	SAT2	0.981							

^a^CR: composite reliability.

^b^AVE: average variance extracted.

^c^CONF: confirmation.

^d^CI: continuance intention.

^e^PEOU: perceived ease of use.

^f^PU: perceived usefulness.

^g^SAT: satisfaction.

**Table 4:** Discriminant validity.

	Confirmation	Continuance intention	Perceived ease of use	Perceived usefulness	Satisfaction
Confirmation	0.943^a^	—^b^	—	—	—
Continuance intention	0.853	0.996^a^	—	—	—
Perceived ease of use	0.177	0.175	0.833^a^	—	—
Perceived usefulness	0.825	0.759	0.192	0.843^a^	—
Satisfaction	0.841	0.857	0.157	0.783	0.980^a^

^a^The square root of the average variance extracted for each construct.

^b^Not available.

### Structure Model Assessment

**Figure 2:**
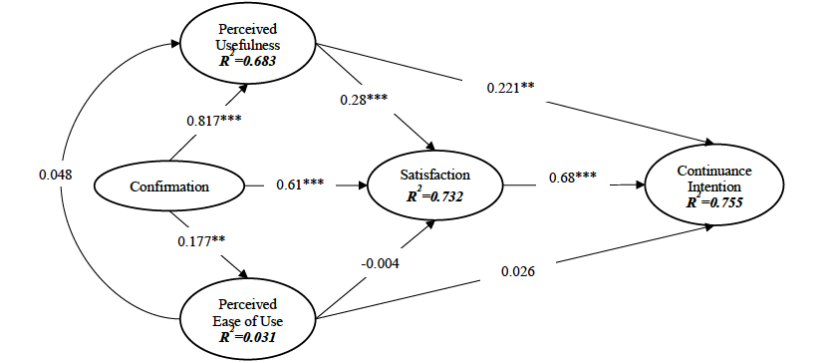
Results of the structure model. *:*P*<.05; **:*P*<.01; *** *P*<.001.

The inner variance inflation factors were below 5, indicating that we were able to avoid construct collinearity in the model [27]. The research model was assessed by evaluating the path coefficients (β) and the coefficients of determination (*R*^2^). The path coefficients and their significance levels, as well as hypothesis outcomes and *R*^2^ values, are shown in Figure 2 and Table 5.

β represents the direct effects of independent variables on dependent variables. The hypotheses that were based on the original ECM (hypotheses 1, 2, 6, 7, and 9) were all supported, while the hypotheses regarding the newly integrated construct—perceived ease of use (hypotheses 3, 4, and 5)—were rejected, except for hypothesis 8. *R*^2^ refers to the amount of explained variance for each endogenous latent variable. The entire model explained 75.5% of the variance in continuance intention and 73.2% of the variance in satisfaction, which was considered substantial.

**Table 5:** Hypothesis test results.

Hypothesis	Path	Coefficient (β)	*P* value	Outcome
Hypothesis 1	Perceived usefulness → continuance intention	.221	.004	Supported
Hypothesis 2	Perceived usefulness → satisfaction	.280	<.001	Supported
Hypothesis 3	Perceived ease of use → continuance intention	−.004	.63	Rejected
Hypothesis 4	Perceived ease of use → satisfaction	.026	.91	Rejected
Hypothesis 5	Perceived ease of use → perceived usefulness	.048	.37	Rejected
Hypothesis 6	Confirmation → perceived usefulness	.817	<.001	Supported
Hypothesis 7	Confirmation → satisfaction	.610	<.001	Supported
Hypothesis 8	Confirmation → perceived ease of use	.177	.007	Supported
Hypothesis 9	Satisfaction → continuance intention	.680	<.001	Supported

### Qualitative Data on Patients’ Expectations

A total of 3 themes were extracted from the 74 answers regarding patients’ specific expectations that CAs failed to meet. The first theme was *personalized interaction* (mentioned 50 times). Instead of the same interaction content and forms of interaction, patients expected to see more personalized conversations that were based on their previous medical histories and visit types. Older participants asked for a voice recognition function and larger font sizes. The second theme was *effective utilization* (mentioned 37 times). Patients expected CAs to have more useful functions, mainly focusing on self-assessments for referrals and self-management for follow-up treatments. The third theme was *clear illustrations on the use and promises of CAs* (mentioned 15 times). In some cases, CAs were easily mistaken as replacements for face-to-face consultations.

## Discussion

### Summary of Findings

This study identified key factors influencing patients' continuance intention toward CAs through an extended ECM. Satisfaction (β=.68; *P*<.001) and perceived usefulness (β=.221; *P*=.004) were significant predictors of continuance intention, with satisfaction being the stronger predictor. Patients' extent of confirmation significantly affected both perceived usefulness (β=.817; *P*<.001) and satisfaction (β=.61; *P*<.001). These findings are consistent with the original ECM as well as the findings of previous research on information system usage (eg, telemedicine and health data reporting platform usage) among patients [32-35]. The confirmation of patients’ expectations has a positive effect on perceived usefulness and satisfaction, and the improvement of perceived usefulness and satisfaction can further enhance patients’ enthusiasm for continuing to use a system.

Our qualitative data shed light on patients’ specific expectations that CAs failed to meet, including personalized interactions, effective utilization, and clear illustrations. Our findings can help administrators and researchers better understand low CA usage rates. After using CAs, if these expectations have not been positively confirmed, perceived usefulness and satisfaction among patients will drop accordingly and result in their unwillingness to continue using CAs.

Although accumulated evidence has shown the significant impact of perceived ease of use on both perceived usefulness and usage intention [15-17], in this study, perceived ease of use turned out to be trivial in the context of CAs. Not coincidentally, some studies on information system usage in hospitals have also shown the insignificant relationship between perceived ease of use and usage intention [36-38]. This result indicates that once CAs prove to be useful and effective, patients will consider it worth their time and effort to learn how to use CAs. However, if CAs are easy to use but cannot collect useful medical histories from patients, patients’ continuance intention will not improve anyway [37].

### Managerial and Public Health Implications

Our findings have important managerial implications. The proposed model provides a feedback channel that administrators can use to gain insight into patients’ actual experiences and expectations. To maximize patients’ satisfaction and continuance intention, efforts should be made toward improving the aspects that patients reasonably expect CAs to have. Offering personalized interactions based on patients’ histories and adding more functions can increase perceived usefulness among patients, while providing clear illustrations on the use and promises of CAs can result in patients having appropriate expectations, which allow for positive postuse confirmation.

### Contributions of This Study

This study contributes to the body of knowledge about the determinants of CA continuance usage. Almost half of the existing literature on CA acceptance, adoption, and usage evaluates a specific CA artifact, while only 21% of studies put the user in the center of attention when investigating the determinants of their acceptance and usage of CAs [39]. Most of these user-focused empirical studies did not draw on specific concepts from theory for their evaluations [40-43], which makes the results hard to compare. The contribution of this paper is 2-fold. From a theoretical point of view, we identified key factors influencing users’ continuance usage intention through a theoretical model. The applicability and validity of the model was empirically tested via a cross-sectional field survey. From a practical point of view, corresponding managerial implications based on the results were proposed to facilitate the successful and continuous development of CAs.

### Study Limitations

This study has several limitations that should be addressed. The digital transformation of CA systems started less than 1 year ago, and the progress of this transformation varies dramatically from hospital to hospital. Therefore, this study was conducted at a hospital with a relatively well-designed system and a larger user base. Further research is needed to confirm our findings in the context of different hospitals and different CAs. Additionally, the sample was not normally distributed in terms of gender and age. However, the data analysis was trustworthy, since a PLS-SEM is capable of producing robust results with restricted sample sizes and data lacking normality. Furthermore, a successful digital transformation in health care is a joint effort, and in terms of CAs, this effort depends not only on patients’ continuance but also on physicians’ utilization and administrators’ management of CAs. A multisource model is required to explore the relationships among the constructs.

### Conclusions

This study intended to identify key factors influencing patients’ continuance intention toward CAs. Satisfaction and perceived usefulness were significant predictors of continuance intention (*P*<.001 and *P*<.004, respectively) and were significantly affected by patients’ extent of confirmation (*P*<.001 and *P*<.001, respectively). Developing a better understanding of patients’ continuance intention can help administrators figure out how to facilitate the effective implementation of CAs. Efforts should be made toward improving the aspects that patients reasonably expect CAs to have, which include personalized interactions, effective utilization, and clear illustrations.

## Appendix

Multimedia Appendix 1 Operationalization of the research variables.

## Abbreviations

AVE: average variance extracted
CA: conversational agent
ECM: expectation-confirmation model
ENT: ear, nose, and throat
PLS-SEM: partial least squares structural equation model
TAM: Technology Acceptance Model
