# Supplementary material for: Investigating Patients' Continuance Intention Toward Conversational Agents in Outpatient Departments: Cross-sectional Field Survey
Source: J Med Internet Res. 2022 Nov 7;24(11):e40681. doi: 10.2196/40681 (PMC9679947; doi:10.2196/40681)
Supplement: Multimedia Appendix 1 [file jmir_v24i11e40681_app1.docx]

## Appendix 1

Operationalization of the research variables.

| **Construct** |  | **Measurement items** | **Source** |
| --- | --- | --- | --- |
| **Continuance Intention** (CI) |  |  |  |
|  | CI1 | I plan to continue using conversational agents(CAs) to describe my symptoms and history beforehand | [11] |
|  | CI2 | I intend to continue using CAs in the future |  |
| **Satisfaction** (S) |  |  |  |
|  |  | How do you feel about your overall experience of CA use: | [11] |
|  | S1 | Very dissatisfied/Very satisfied |  |
|  | S2 | Very displeased/Very pleased |  |
| **Perceived Usefulness** (PU) |  |  |  |
|  | PU1 | CAs allowed me to describe my symptoms and history beforehand | [11] |
|  | PU2 | Using CAs improved the communication efficiency with doctors during face-to-face consultation |  |
|  | PU3 | Overall, conversational agents were useful during the visit |  |
| **Perceived ease of Usefulness** (PEOU) |  |  |  |
|  | PEOU1 | Learning to use CAs was easy for me | [17] |
|  | PEOU2 | My interaction with CAs was clear and understandable |  |
|  | PEOU3 | Overall, I found that the Dialogue platform is easy to use |  |
|  |  |  |  |
| **Confirmation** (CONF) |  |  |  |
|  | CONF1 | My experience of CA use was better than what I expected | [11] |
|  | CONF2 | The benefits provided by CAs was better than what  I expected |  |
|  | CONF3 | Overall, most of my expectations from using CAs were  confirmed |  |
